# Meiotic cohesin-based chromosome structure is essential for homologous chromosome pairing in *Schizosaccharomyces pombe*

**DOI:** 10.1007/s00412-015-0551-8

**Published:** 2015-10-28

**Authors:** Da-Qiao Ding, Atsushi Matsuda, Kasumi Okamasa, Yuki Nagahama, Tokuko Haraguchi, Yasushi Hiraoka

**Affiliations:** Advanced ICT Research Institute Kobe, National Institute of Information and Communications Technology, 588-2 Iwaoka, Iwaoka-cho Nishi-ku, Kobe, 651-2492 Japan; Graduate School of Frontier Biosciences, Osaka University, 1-3 Yamadaoka, Suita, 565-0871 Japan

**Keywords:** Meiosis, Homologous chromosome, Cohesin, Pairing, Recombination, Fission yeast

## Abstract

Chromosome structure is dramatically altered upon entering meiosis to establish chromosomal architectures necessary for the successful progression of meiosis-specific events. An early meiotic event involves the replacement of the non-SMC mitotic cohesins with their meiotic equivalents in most part of the chromosome, forming an axis on meiotic chromosomes. We previously demonstrated that the meiotic cohesin complex is required for chromosome compaction during meiotic prophase in the fission yeast *Schizosaccharomyces pombe*. These studies revealed that chromosomes are elongated in the absence of the meiotic cohesin subunit Rec8 and shortened in the absence of the cohesin-associated protein Pds5. In this study, using super-resolution structured illumination microscopy, we found that Rec8 forms a linear axis on chromosomes, which is required for the organized axial structure of chromatin during meiotic prophase. In the absence of Pds5, the Rec8 axis is shortened whereas chromosomes are widened. In *rec8* or *pds5* mutants, the frequency of homologous chromosome pairing is reduced. Thus, Rec8 and Pds5 play an essential role in building a platform to support the chromosome architecture necessary for the spatial alignment of homologous chromosomes.

## Introduction

Meiosis is an important process for sexually reproducing eukaryotic organisms, generating inheritable haploid gametes from a parental diploid cell. During this process, the pairing of homologous chromosomes results in recombination-mediated physical links between them that are essential for the correct segregation of meiotic chromosomes. Understanding the mechanisms involved in the pairing and recombination of homologous chromosomes is clinically important because chromosome missegregation during meiosis is a major cause of human miscarriage and developmental abnormalities (Nagaoka et al. 2012).

During the pairing process, each homologous pair of chromosomes is selectively aligned. It has been suggested that a “bouquet” arrangement of chromosomes, in which chromosomes are bundled at the telomeres to form a polarized configuration, contributes to the pairing of homologous chromosomes by spatially aligning them (Zickler and Kleckner 1998; Scherthan 2001; Chikashige et al. 2007). The fission yeast *Schizosaccharomyces pombe* exhibits a striking example of the bouquet arrangement. In this organism, the nucleus elongates and moves back and forth between the cell ends during meiotic prophase while telomeres remain clustered at the leading edge of the moving nucleus (Chikashige et al. 1994; Ding et al. 1998). This elongated nucleus is generally called a “horsetail” nucleus. Several mutants defective in telomere clustering and oscillatory chromosome movements exhibit a reduced frequency of homologous recombination, suggesting that these activities play a role in the pairing of homologous chromosomes (Chikashige and Hiraoka 2001; Cooper et al. 1998; Kanoh and Ishikawa 2001; Nimmo et al. 1998; Shimanuki et al. 1997; Yamamoto et al. 1999; Davis and Smith 2006). Live cell imaging of meiotic cells demonstrated that telomere clustering and oscillatory chromosome movements spatially align homologous chromosomes in the early stages of meiotic prophase to promote their association during the pairing process (Ding et al. 2004). On chromosomal arms, these contacts are subsequently stabilized by a pathway dependent on Rec12 (Spo11 in budding yeast *Saccharomyces cerevisiae*), a protein which catalyzes the formation of DNA double-strand breaks (DSB) that initiate homologous recombination in meiosis (DeVeaux and Smith 1994; Ding et al. 2004; Keeney et al. 1997; Nabeshima et al. 2001; reviewed in Ding et al. 2010). However, inter-homolog associations at centromeres gradually increase during the horsetail stage with similar dynamics observed for both wild type and *rec12*^-^ mutant cells, suggesting that pairing at centromeres is stabilized by a pathway that is independent of DSB formation (Ding et al. 2004). Additionally, recombination-independent pairing of homologous chromosomes at chromosomal arms has also been demonstrated in which pairing is mediated by non-coding RNA that accumulates at the *sme2* gene locus (Ding et al. 2012).

Meiotic cohesins are essential for sister chromatid cohesion and are the main component of axial elements, which subsequently form lateral elements of the synaptonemal complex (SC) (Page and Hawley 2003). The mitotic cohesin complex in *S. pombe* comprises Psm1, Psm3, Rad21, and Psc3. Upon entering meiosis, the majority of Rad21 and Psc3 are replaced by meiosis specific components Rec8 and Rec11, respectively (Parisi et al. 1999; Watanabe and Nurse 1999; Yokobayashi et al. 2003). In addition to the core cohesin complex, a conserved cohesin-associated protein called Pds5 (Spo76 in *Sodaria*; BimD in *Aspergillus*) is involved in the maintenance of sister chromatid cohesion (van Heemst et al. 1999; Hartman et al. 2000; Panizza et al. 2000; Tanaka et al. 2001) and also affects chromosome morphology in meiotic prophase (Storlazzi et al. 2008; Jin et al. 2009; van Heemst et al. 2001). We previously reported that chromosomes become significantly less compacted in the absence of Rec8, whereas the loss of Pds5 results in Rec8-dependent over-compaction (Ding et al. 2006). Unlike many other organisms, *S. pombe* does not assemble canonical SC structures and no obvious chromosome condensation occurs at the horsetail stage. However, *S. pombe* forms the so-called linear elements (LinEs), which are evolutionally related to the axial/lateral elements of the SC (Bahler et al. 1993; Loidl 2006; Lorenz et al. 2004; Fowler et al. 2013). The components of LinEs are required for DSB formation and recombination (Davis et al. 2008; Ellermeier and Smith 2005; Estreicher et al. 2012; Fowler et al. 2013). It has been previously demonstrated that meiotic cohesin promotes LinEs formation: Rec8 is required for the localization of Rec11 to meiotic prophase chromosomes (Ding et al. 2006), and phosphorylated Rec11 is required for the assembly of LinEs (Sakuno and Watanabe 2015).

Although the role of meiotic cohesins in meiotic prophase chromosome compaction has been reported, their involvement in homologous chromosome pairing and chromosome structure remains unclear. In this study, we investigated the role of meiotic cohesin Rec8 and Pds5 in homologous chromosome pairing in *S. pombe*. We also studied chromosome and cohesin-axis structures in live cells during meiotic prophase by super resolution three-dimensional structured illumination microscopy (3D-SIM) (Schermelleh et al. 2008). Here, we report the establishment of meiotic cohesin-mediated chromosome structures and their roles in homologous chromosome pairing.

## Materials and methods

### Strains and culture

The *S. pombe* strains used in this study are listed in Table 1. Visualization of chromosome loci at *ade8*, *cen2*-proximal, and *sme2* using the lac repressor (lacI-GFP)/*lac* operator (*lacO*) recognition system was described previously (Ding et al. 2004; Ding et al. 2012). The *rec8*^-^ and *pds5*^-^ mutants as well as Rec8-GFP fusion proteins used in this study were described previously (Watanabe and Nurse 1999). Histone H2B-GFP and H3-mCherry fusion proteins were constructed as described in Matsuda et al. (2015).

**Table 1:** Strain list

Strain	Genotype
Fig. 1a, b	
MK161	*h* ^*90*^ *ade6*-*149 leu1*-*32 lys1*-*131 ura4*-*D18* ∆*rec8*::*ura4* ^+^ *cen2*[::*ura4* ^+^-*kan* ^*r*^-*lacOp*] *his7* ^+^::*lacI*-*GFP*
CT050-2B	*h* ^*90*^ *leu1*-*32 lys1*-*131 ura4*-*D18 ade8*[::*ura4* ^+^-*kan* ^*r*^-*lacOp*] *his7* ^+^::*lacI*-*GFP*
AY208-4A	*h* ^-^ *leu1*-*32 ura4*-*D18* ∆*rec8*::*ura4* ^+^ *ade8*[::*ura4* ^+^-*kan* ^*r*^-*lacOp*] *his7* ^+^::*lacI*-*GFP*
AY266-5A	*h* ^+^ *lys1*-*131 ura4*-*D18* ∆*rec8*::*ura4* ^+^ *ade8*[::*ura4* ^+^-*kan* ^*r*^-*lacOp*] *his7* ^+^::*lacI*-*GFP*
YY297-3D	*h* ^-^ *leu1*-*32 ura4*-*D18* ∆*pds5*::*LEU2 ade8*[::*ura4* ^+^-*kan* ^*r*^-*lacOp*] *his7* ^+^::*lacI*-*GFP*
YY297-10D	*h* ^+^ *leu1*-*32 lys1*-*131 ura4*-*D18* ∆*pds5*::*LEU2 ade8*[::*ura4* ^+^-*kan* ^*r*^-*lacOp*] *his7* ^+^::*lacI*-*GFP*
CT2112-2	*h* ^*90*^ *ade6*-*216 leu1*-*32 lys1*-*131 ura4*-*D18 cen2*[::*ura4* ^+^-*kan* ^*r*^-*lacOp*] *his7* ^+^::*lacI*-*GFP*
YY307-1A	*h* ^*90*^ *ade6*-*210 leu1*-*32 lys1*-*131 ura4*-*D18* ∆*pds5*::*LEU2 cen2*[::*ura4* ^+^-*kan* ^*r*^-*lacOp*] *his7* ^+^::*lacI*-*GFP*
YY548-13C	*h* ^*90*^ *ade6*-*216 leu1*-*32 ura4*-*D18 sme2proxy*[::*ura4* ^+^-*kan* ^*r*^-*lacOp*] *his7* ^+^::*lacI*-*GFP*
YY553-11B	*h* ^*90*^ *ade6*-*216 leu1*-*32* ∆*rec8*::*ura4* ^+^ *sme2proxy*[::*ura4* ^+^-*kan* ^*r*^-*lacOp*] *his7* ^+^::*lacI*-*GFP*
YW073-2D	*h* ^*90*^ *ade6*-*216 leu1*-*32 ura4*-*D18* ∆*pds5*::*LEU2 sme2proxy*[::*ura4* ^+^-*kan* ^*r*^-*lacOp*] *his7* ^+^::*lacI*-*GFP*
Fig. 1c	
YY350-4A	*h* ^-^ *ade8 trp1*
YY350-4D	*h* ^+^
YY312-6C	*h* ^+^ *his2 leu1*-*32* ∆*pds5*::*LEU2*
YY355-13A	*h* ^-^ *ade8 trp1 leu1*-*32 ura4*-*D18* ∆*pds5*::*LEU2*
YW262-7A	*h* ^-^ ∆*rec8*::*ura4* ^+^
YW262-10B	*h* ^+^ *ade8 trp1* ∆*rec8*::*ura4* ^+^
AY153-19B	*h* ^+^ *his2 ade6*-*469*
AY161-2C	*h* ^-^ *ura4*-*D18 ade6*-*M26*
AY180-17D	*h* ^-^ *leu1*-*32 fur1*
YY317-1A	*h* ^+^ *his2 ade6*-*469 leu1*-*32* ∆*pds5*::*LEU2*
YY318-5B	*h* ^-^ *ura4*-*D18 ade6*-*M26* ∆*pds5*::*LEU2*
YY319-3A	*h* ^-^ *leu1*-*32 fur1* ∆*pds5*::*LEU2*
Fig. 2a	
YAM033	*h* ^*90*^ *leu1*-*32 lys1* ^+^[::*hta1* ^+^-*htb1* ^+^-*GFP*]
YAM035	*h* ^*90*^ *leu1*-*32* ∆*pds5*::*LEU2 aur1* ^*r*^[::*hta1* ^+^-*htb1* ^+^-*GFP*]
YAM036	*h* ^*90*^ *l ura4*-*D18* ∆*rec8*::*ura4* ^+^ *aur1* ^*r*^[::*hta1* ^+^-*htb1* ^+^-*GFP*]
Fig. 2b	
PY183	*h* ^*90*^ *ade6*-*216 leu1*-*32 rec8* ^+^::*GFP*-*kan* ^*r*^
YY286-1B	*h* ^-^ *ade6*-*216 leu1*-*32 ura4*-*D18* ∆*pds5*::*LEU2 rec8* ^+^::*GFP*-*kan* ^*r*^
YY286-3A	*h* ^+^ *his2 leu1*-*32 lys1*-*131 ura4*-*D18* ∆*pds5*::*LEU2 rec8* ^+^::*GFP*-*kan* ^*r*^
Fig. 3	
YW267-1	*h* ^*90*^ *ade6*-*216 leu1*-*32 rec8* ^+^::*GFP*-*kan* ^*r*^ *aur1* ^*r*^[::*hhf2* ^+^-*hht2* ^+^-*mCherry*]

### Live cell analysis of homologous chromosome pairing

For deconvolution microscopy, a DeltaVision Elite microscope (GE Healthcare, Buckinghamshire, UK) with an objective lens 60× PlanApo NA 1.4 Oil SC (Olympus) set up in a temperature-controlled room was used (Haraguchi et al. 1999). Data analysis was carried out by using SoftWoRx software on the DeltaVision system (Agard et al. 1989; Chen et al. 1996).

Cells were grown on solid YES medium at 33 °C. To induce meiosis, the cells were transferred to solid ME medium and incubated at 26 °C for about 12 h. They were then suspended in EMM-N medium supplemented with the appropriate amino acids for live observations. The cell suspension was placed in a 35-mm glass-bottom culture dish (MatTek Corp., Ashland, MA, USA) coated with 0.2 % (*w*/*v*) lectin. The behavior of GFP-labeled chromosomal loci in meiotic cells was examined at 26 °C as described previously (Ding et al. 2004). A set of images from 15 focal planes with 0.3-μm intervals was taken every 5 min. At least 20 individual zygotes were observed for each experiment. Data analysis to determine the frequency of pairing was performed as previously described (Ding et al. 2004). Briefly, we defined the period from the end of karyogamy to the end of oscillatory nuclear movements as the horsetail stage and divided the horsetail stage in each zygote cell equally into five substages (each substage is about 25 min on average). We then measured the distance between two homologous loci in 3D space and counted the number of time points at which two homologous loci were associated with each other in each substage. The measured frequency of pairing was then plotted as a time course. We defined homologous loci as being “paired” when the distance between the center of the GFP signals was equal to or less than 0.35 μm (the diameter of the GFP signal) (i.e., when the signals overlapped or were linked with one another). In averaging the distances, a distance equal to or less than 0.35 μm was regarded as 0.35 μm.

### Analysis of recombination frequency

Strains bearing appropriate genetic markers were crossed and random spore analysis was used to examine the frequency of intergenic or intragenic recombination. The sporulated zygotes on ME plates (26 °C, 3 days) were treated with 0.2 % glusalase at 37 °C for 3 h to release the spores, and then 30 % ethanol was added for 15 min to kill the remaining non-spore cells. The spores were growth on YES medium (with supplements when required), and the spore colonies formed after 2~3 days were replicated to plates with selective medium. More than 400 colonies were checked for each cross in the intergenic recombination assays and more than 5000 colonies in the case of the intragenic ones; each experiment was repeated three times. The recombination frequency was calculated as the percentage of recombinant spore among total spores.

### SIM analysis of live cells in meiotic prophase

For 3D-SIM imaging, we used a DeltaVision|OMX microscope version 3 (GE Healthcare) with an objective lens 100× UPlanSApo NA1.40 Oil (Olympus, Tokyo, Japan). For live cell SIM, cells in EMM2-N attached to glass-bottom dishes coated with lectin were imaged with immersion oil with a refractive index of 1.522. Live cell SIM reconstruction was performed by using the softWoRx software (GE Healthcare) with a wiener filter constant of 0.012. To cover the entire nucleus, a set of 17 optical sections were taken at 0.125-μm focus intervals. For simultaneous observations of H3-mCherry and Rec8-GFP, a set of nine optical sections were taken. Priism suite (http://msg.ucsf.edu/IVE/) was used for the correction of chromatic aberrations, camera alignment, and for the measurement of chromosome width and Rec8-axis. We selected cells that were undergoing horsetail movements and had fluorescent signals strong enough to perform the 3D-SIM analysis on and not specify the stage of prophase.

## Results and discussion

### Rec8 and Pds5 are required for the alignment and pairing of homologous chromosomes during meiotic prophase

Defective homologous pairing in Rec8 mutant cells was reported by fluorescence in situ hybridization analysis of spread chromosomes in *S. pombe* (Molnar et al. 1995) and in the nematode *C. elegans* (Pasierbek et al. 2001). In *S. cerevisiae*, Rec8 has been shown to be required for pairing in a cohesion-independent way (Brar et al. 2009), and Pds5 is required for synapsis of homologous chromosomes in a Rec8-dependent manner (Jin et al. 2009). On the other hand, meiotic prophase-specific horsetail nuclear movements were observed in *rec8*^-^ and *pds5*^-^ mutant cells and microtubule dynamics were indistinguishable from those observed in wild-type cells (data not shown), although the shape of the horsetail nucleus appeared aberrant since chromatin structures were altered (Ding et al. 2006). Telomere clustering was also normal in *rec8*^-^ cells (Molnar et al. 1995) and in most cells of the *pds5*^-^ mutant, with the rare observation that in a few *pds5*^-^ cells, one or two telomeres occasionally separated from the main telomere cluster (data not shown).

**Fig. 1:**
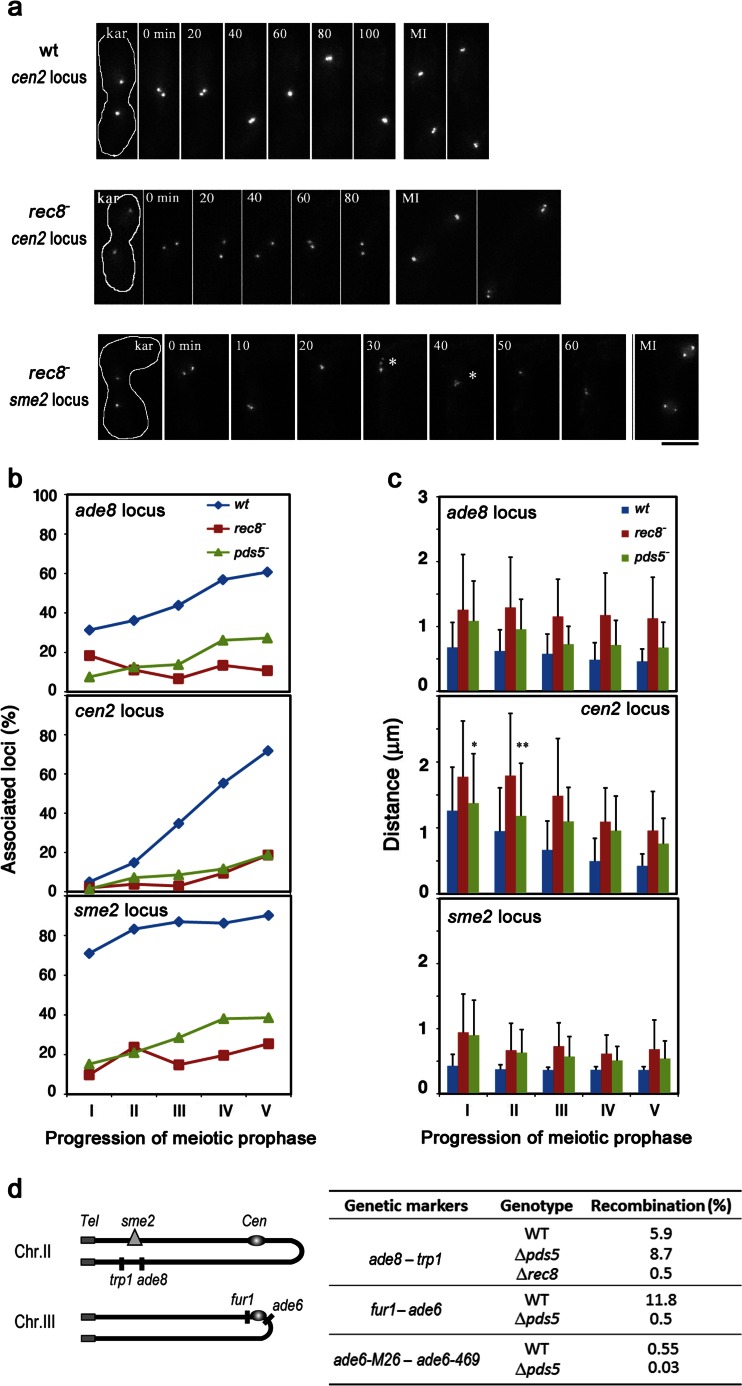
Homologous chromosome association and recombination in *rec8* ^-^ and *pds5* ^-^ mutants. **a** Selected time-lapse images of live cell observations of the *cen2* locus in a wild-type cell (*upper panel*) and in a *rec8* ^-^ cell (*middle panel*). The *sme2* locus in a *rec8* ^-^ cell is shown in the *bottom panel*. Labels “kar” and “MI” represent karyogamy and meiosis I, respectively. *Numbers* indicate the time in minutes after nuclear fusion. The *asterisks* indicate precocious separation of sister chromatids. *Bar* = 5 μm. **b** Time course of the homologous association frequency at *ade8*, *cen2*, and *sme2* loci during meiotic prophase in wild type (*blue diamond*), *rec8* ^-^ (*red square*), and *pds5* ^-^ (*green triangle*) cells. Meiotic prophase was divided equally into five substages (I–V) from karyogamy to the end of the horsetail movement for each cell. For each strain, 20 to 30 cells were examined as previously described (Ding et al. 2004). **c** Average distance between homologous chromosome loci. The inter-homolog distance was measured from 3D image stacks for each cell. The average distances are shown at the *ade8* (*upper panel*), *cen2* (*middle panel*), and *sme2* (*bottom panel*) loci in wild type (*blue*), *rec8* ^-^ (*red*), and *pds5* ^-^ (*green*) cells. The *asterisks* indicate no significant difference with wild-type cells (*p = 0.9, **p = 0.07). The rest of the data show significant differences with *p* values less than 0.001. **d** Homologous recombination in wild type and mutant cells. The average frequency of recombination in random spore analyses from three independent experiments is shown. Chromosomal positions of the genetic loci examined are schematically shown in the *left panel*

To evaluate the contribution of the meiotic cohesin components Rec8 and Pds5 to the pairing process over time, we directly observed the dynamics of pairing between homologous chromosomal loci that were marked with a *lacO*/LacI-GFP tag (Ding et al. 2004) (Fig. 1a). In cells of the *rec8*^-^ or *pds5*^-^ mutant, the association of homologous chromosomes was impaired at both the arm (*ade8*) and centromere (*cen2*) regions (Fig. 1b). The average distance between homologous loci at both chromosomal regions was significantly greater in *rec8*^-^ cells than in wild-type cells (*p* < 0.001) at every stage of meiotic prophase (Fig. 1c), indicating that the proper spatial alignment of homologous chromosomes was not achieved in *rec8*^-^ cells. In *pds5*^-^ cells, the inter-homolog distance was significantly greater than in wild-type cells, except the first stage for the *cen2* locus in which the average distance was comparable to wild-type cells (Fig. 1c, *cen2* locus, labeled with asterisks). Also, the distance in *rec8*^-^ mutants was usually greater than in *pds5*^-^ mutants, suggested that Rec8 influences chromosome alignment more prominently than Pds5. Additionally, arm and centromere regions displayed different behaviors during meiotic prophase progression, in which the decrease in inter-homolog distance at centromeres is more dramatic than that at the arm region even in *rec8*^-^ or *pds5*^-^ cells (Fig. 1c). These data suggest that arm regions are more dependent on cohesin-dependent chromosome pairing than are centromeres. We previously showed mild sister chromatid cohesion defects at the *ade8* locus in *rec8*^-^ and *pds5*^-^ cells, while cohesion remained at wild-type levels at the *cen2* region (Ding et al. 2006). Nevertheless, pairing in these mutants at both loci was dramatically decreased. These observations suggest that Rec8 and Pds5 contribute to homologous chromosome pairing mostly through the process of homologous chromosome alignment independent of sister cohesion. A similar phenomenon has been found in *S. cerevisiae*, in which the role of Rec8 in pairing can be separated from its role in sister chromatic cohesion (Brar et al. 2009; Jin et al. 2009).

Homologous recombination is strongly inhibited in *rec8*^-^ mutants (De Veaux et al. 1992; Krawchuk and Wahls 1999; Parisi et al. 1999). Because homologous chromosome pairing was defective in the absence of Pds5, we also investigated the effect of Pds5 on homologous recombination. In *pds5*^-^ mutants, both intergenic and intragenic homologous recombination was decreased to about 5 % of the wild-type level at the *ade6* locus. However, no reduction in recombination was found at the *trp1* and *ade8* interval (Fig. 1d) despite defective pairing and alignment of homologous chromosomes at this region in *pds5*^-^ mutants (Fig. 1b, c). We thus checked the recombination rate at this region in *rec8*^-^ cells. It was reported that recombination is reduced in *rec8*^-^ cells in the interval *arg4*-*tpr1* (*arg4*-*tpr1* is a large interval that covers the *ade*8-*trp1* interval) (Ellermeier and Smith 2005). Consistent with the published data, recombination between *ade8* and *trp1* in *rec8*^-^ cells was reduced to about 10 % of the wild-type level (Fig. 1d). Thus, unlike Rec8, the requirement of Pds5 for homologous recombination might be limited to centromeric regions. Given the opposite impact on chromosome compaction by Rec8 and Pds5 (Ding et al. 2006), it is likely that these two proteins contribute to recombination differently. Defective recombination at specific chromosomal regions (i.e., stronger defects at centromere-proximal regions) has also been reported in *rec8*^-^ and *rec11*^-^ cells (De Veaux et al. 1992; Krawchuk and Wahls. 1999). Further investigations of recombination rates at other chromosomal regions and on different chromosomes in *pds5*^-^ cells will provide a more complete conclusion. The apparent discrepancy between recombination and pairing frequencies observed at arm regions may reflect the fact that recombination reactions are much faster than the process of pairing. Possibly, recombination is completed during a transient contact of homologous loci while pairing analysis can detect only stable contacts that occur during a relatively slow process. In addition, homologous pairing and recombination are defective in the Pds5 mutant background in both *Sordaria* and *S. cerevisiae* (van Heemst et al. 1999; Jin et al. 2009) in consistent with our result in *S. pombe*.

### Rec8 and Pds5 are required for RNA-mediated homologous pairing during meiotic prophase

We previously reported that the *sme2* locus exhibits a robust level of homologous pairing in early meiotic prophase in *S. pombe*. This robust pairing is mediated by meiosis-specific non-coding RNA and depends on telomere clustering (Ding et al. 2012). We therefore examined whether this robust pairing requires meiotic cohesins. Our results show that homologous pairing at the *sme2* locus was impaired in the absence of Rec8 or Pds5 (Fig. 1a, b). In both mutants, the spatial alignment of homologous *sme2* loci was defective (Fig. 1c). In the absence of Rec8, precocious sister chromatid separations were observed at the *sme2* locus in 13 % of cells during meiotic prophase (Fig. 1a, indicated by the asterisks), a result similar to the *ade8* locus. Thus, meiotic cohesins are also required for pairing even at the RNA-mediated robust pairing site. Taken together, these results suggest that chromosome architectures organized by meiotic cohesins are a prerequisite for the alignment of homologous chromosomes.

### Lack of Rec8 or Pds5 alters meiotic prophase chromosome architectures

We have shown that both Rec8 and Pds5 are necessary for promoting the homologous chromosome pairing in *S. pombe*. Rec8 along the chromosome axis may directly promote interactions between homologous chromosomes, and a reduced amount of Rec8 in the absence of Pds5 would be insufficient for the process. However, since the spatial alignment of homologous chromosomes occurs prior to direct contact between them and this process was impaired in *rec8*^-^ and *pds5*^-^ cells, we hypothesized that Rec8, Pds5, and other meiotic cohesins provide a platform to support proper chromosome architecture.

**Fig. 2:**
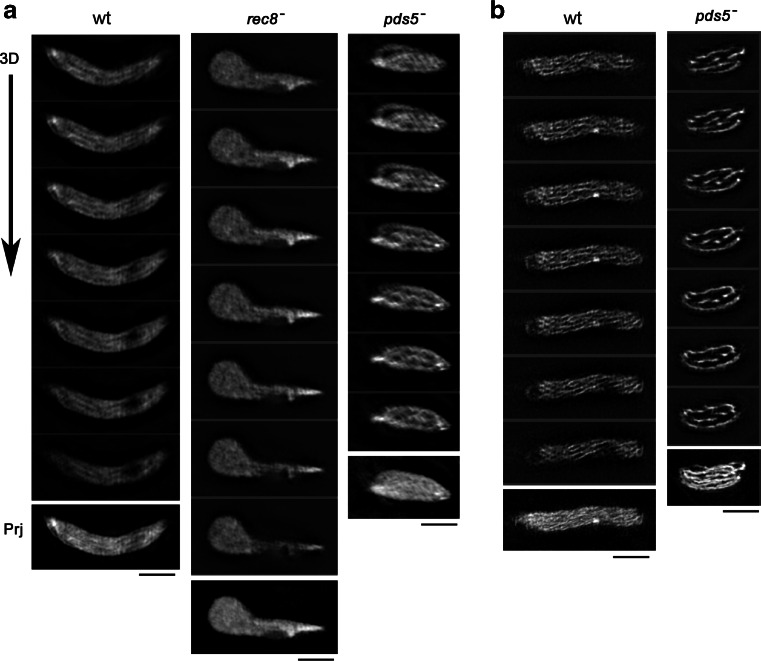
3D-SIM live imaging reveals chromosome morphology and the Rec8-axis. Selected continuous Z-focus planes obtained at 0.125-μm focus intervals in living cells during meiotic prophase are shown (“3D”). Maximum-intensity projections through the entire nucleus (17 focus planes) are shown beneath the panel of 3D images (“Prj”). **a** H2B-GFP in a wild type, *rec8* ^-^, and *pds5* ^-^ mutant cell. **b** Rec8-GFP in a wild type and *pds5* ^-^ mutant cell. *Bars* = 2 μm

**Fig. 3:**
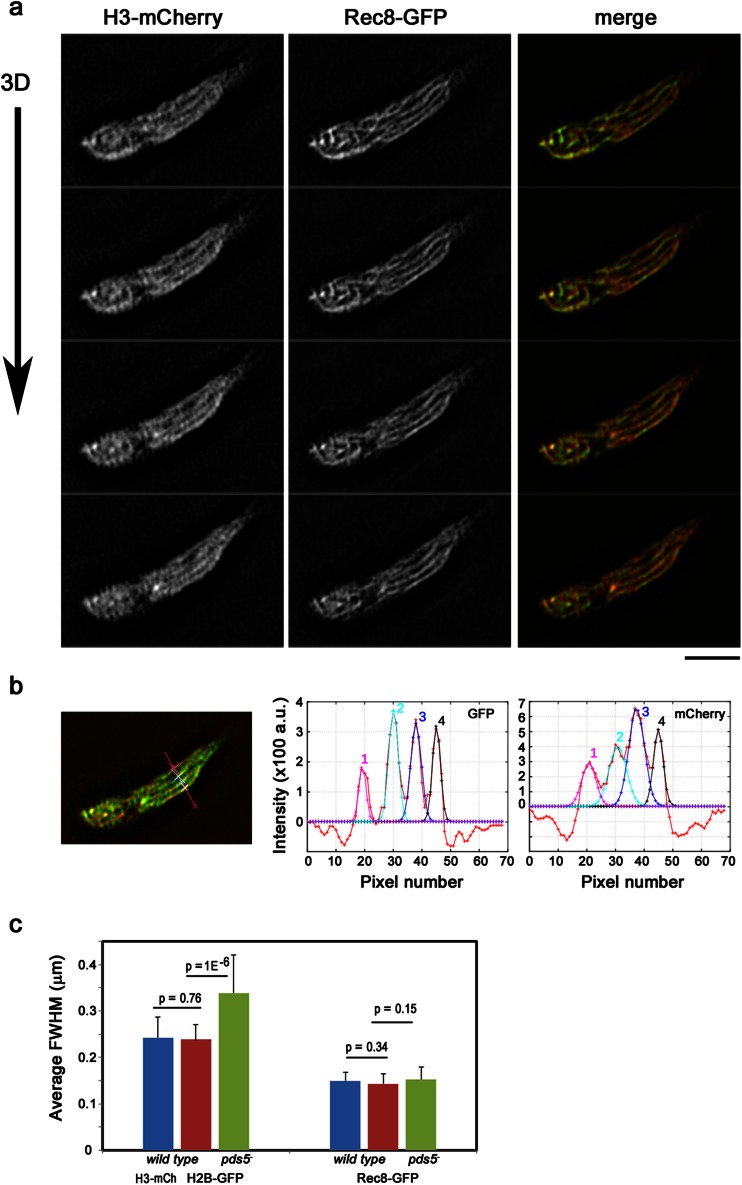
Quantitation of chromosome and cohesin axis width by 3D-SIM imaging. **a** A representative image of histone H3-mCherry and Rec8-GFP simultaneously captured using two cameras (*Bar* = 2 μm). **b** Measurements of the width of chromosomes and Rec8 axes. A line was drawn manually on a section of the image (*red line on the left panel*), and the pixel intensity along the line was plotted (*red line in the middle and right panels*, for Rec8-GFP and histone H3-mCherry, respectively). The pixel intensity profile contains four peaks which are numbered in the middle and right panels for Rec8-GFP and histone H3-mCherry, respectively. Each separate peak in intensity was fitted with a Gaussian distribution with the assumption that the median of the whole 3D stack represented the base intensity with no fluorescence. The full width at half maximum (FWHM) for each numbered peak was calculated from the Gaussian profile. **c** Average FWHM with standard deviation of H2B-GFP (*red and green*) or H3-mCherry (*blue*) labeled chromosomes and Rec8-GFP labeled cohesin axes in wild type and *pds5* ^-^ mutants. Samples in blue were imaged at the same time in the same cell, while red and green samples were imaged separately

We previously demonstrated that chromosomes are significantly elongated in the absence of Rec8 and shortened in the absence of Pds5. The chromosome shortening in the *pds5*^-^ mutant depends on the presence of Rec8 (Ding et al. 2006). To determine the role of meiotic cohesin in building proper chromosome architecture, we performed studies using the super-resolution SIM system for live cells during meiosis. In these experiments, histone H2B-GFP (Fig. 2), H3-mCherry (Fig. 3a, for double labeling of histone and Rec8 in the same cell) and Rec8-GFP were used to visualize chromosomes and Rec8 in live cells. In wild-type cells, each chromosome is clearly distinguished as filamentous structures aligned parallelly in the telomere-clustered horsetail nucleus (Fig. 2a). However, chromosome filaments completely disappeared and only uniform fluorescent signal in the horsetail nucleus was observed in *rec8*^-^ mutant cells (Fig. 2a). In contrast to *rec8*^-^ cells, chromosome filaments were even wider in *pds5*^-^ cells than in wild-type cells (Fig. 2a). These chromosome morphologies appeared after karyogamy and persisted during the entire horsetail stage for approximately 2 h and no striking differences occurred although the fluorescent intensity increased during the progression through meiotic prophase. Thus, formation of a properly organized chromosome filament requires Rec8 and Pds5 in *S. pombe*.

Rec8 also formed filaments in the horsetail nucleus (Fig. 2b, wt). Double labeling of Rec8 and chromosomes using Rec8-GFP and histone H3-mCherry, respectively, showed Rec8 localization along the entire length of the chromosome: the Rec8 axis appears sharper or clearer than the chromosome with histone labeling in both wild type and the *pds5*^-^ mutant (Fig. 2b, Fig. 3a), indicating that Rec8 forms the central core of the chromosome axis. We then estimated the apparent width of the chromosome and the Rec8 axis by 3D-SIM imaging analysis (Fig. 3b). In wild-type cells, the width of the Rec8 axis was estimated to about 145 nm and the width of the histone axis was about 240 nm (Fig. 3c). No statistically significant difference was found between H2B-GFP and H3-mCherry-labeled chromosomes (*p* = 0.76) or Rec8-GFP in different strains (*p* = 0.34) (Fig. 3c). Thus, chromosomes are apparently wider than the Rec8 axis during meiotic prophase. These results suggest that the axis-loop model of meiotic chromatin is applicable to *S. pombe*, in which chromatin loops are bundled along the cohesin axis (Zickler and Kleckner 1999).

In the absence of Pds5, chromosomes are wider and shorter than in wild-type cells (Fig. 2a, *pds5*^-^). The chromosome width was about 340 nm in *pds5*^-^ mutants, which is 1.4-times wider than that in wild-type cells (Fig. 3c). Despite having wider chromosomes, the width of the Rec8 axis was about 152 nm in *pds5*^-^ mutants and was similar to wild-type cells (*p* = 0.15) (Fig. 3c). In a previous study, we found that *pds5*^-^ mutants had reduced Rec8 binding to chromosomes and displayed a 2.2-times shortening of the longitudinal length of the chromosome (Ding et al. 2006). The wider chromosomes in *pds5*^-^ mutants suggest that longitudinal shortening of the chromosomes may be the result of an increase of out-of-axis chromatin when the cohesin anchor points are reduced along the chromosome axis. Regulating the proper compaction of chromosomes in meiotic prophase by Rec8 and Pds5 has also been found in *Sordaria* and in *S. cerevisiae* (Storlazzi et al. 2008; Jin et al. 2009), indicating conserved roles of meiotic cohesins. Rec8 and Pds5 may coordinately control the proper organization of a meiotic cohesin axis specifically required for meiosis events. In *Sordaria*, destabilization of Pds5-decorated axial elements is dependent on DSBs (Storlazzi et al. 2003). Further studies of relationship of DSB formation and chromosome axis morphology may be interesting in understanding the whole picture of meiotic cohesins in homologous chromosome pairing and recombination.

In contrast to *S. pombe*, multiple non-SMC subunits of meiotic cohesin have been found in mammals (Lee and Hirano 2011). A recent study showed that one of the two meiotic cohesins (i.e., RAD21L rather than REC8) plays a critical role in homologous chromosome pairing in mice. RAD21L may promote homologous recognition and alignment through its influence on chromosome architecture (Ishiguro et al. 2015). Our results using live-cell 3D-SIM show that the loss of the meiotic cohesin component Rec8 and the cohesin accessory protein Pds5 changes meiotic prophase chromosome architecture. Thus, meiotic cohesins play a critical role in establishing meiosis-specific chromosome architectures and perturbation of these structures may lead to defective homologous chromosome recognition and pairing.

## Conclusions

We used live-cell imaging of meiotic chromosomes in *S. pombe* to demonstrate that homologous chromosome pairing is significantly impaired in the absence of meiotic cohesin components both at chromosome arm regions and at centromeric regions. Meiotic cohesin is also required for the RNA-mediated robust pairing at the *sme2* locus. Super-resolution microscopy with 3D-SIM demonstrated that the meiotic chromosome structure is altered in the absence of Rec8 or Pds5, a meiotic cohesin subunit. Thus, meiotic cohesins form a foothold for meiotic chromosomes and provide a physical platform for the recognition, pairing, and recombination of homologous chromosomes.
